# Supplementary material for: Cell specific delivery of modified mRNA expressing therapeutic proteins to leukocytes
Source: Nat Commun. 2018 Oct 29;9:4493. doi: 10.1038/s41467-018-06936-1 (PMC6206083; doi:10.1038/s41467-018-06936-1)
Supplement: Supplementary file 1 — Supplementary Information [file 41467_2018_6936_MOESM1_ESM.pdf]

Cell Specific Delivery of Modified mRNA Expressing Therapeutic Proteins to Leukocytes

Veiga et al.

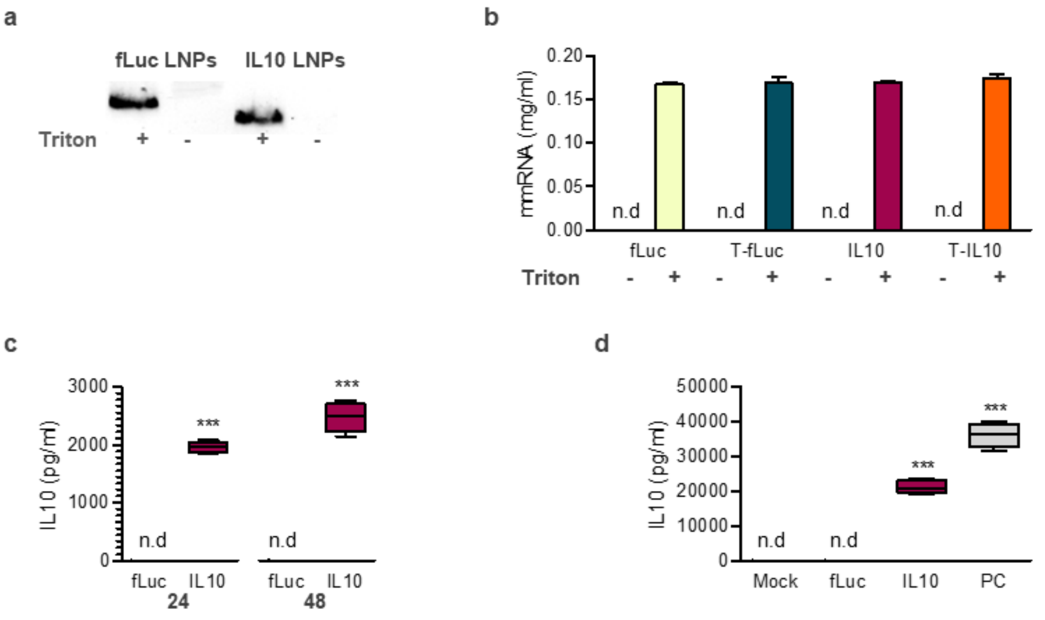

Supplementary Figure 1. **LNPs and tLNPs characterization**

(**a**) Agarose gel electrophoresis to detect free mmRNAs released from Triton-permeabilized or intact LNP. (**b**) Ribogreen quantification of medium's mmRNA concentration released from Triton-permeabilized or intact LNP and tLNPs. (**c-d**) ELISA quantification of in-vitro IL10 expression in RAW 264.7 cells incubated for 24-48 hours (c) and 24 (d) with mmRNA LNPs or transfection reagent as a positive control, 5 µg/ml mmRNA. Data are mean ± s.d. (b) and IQR with a median center line and min to max error bars (c-d), n = 4, * denote $p < 0.05$, ** denote $p < 0.01$, *** denote $p < 0.001$. Statistical analysis in (c) was calculated using two-sided Student's *t*-test. Statistical analysis in (d) was evaluated using one-way ANOVA with Dunnett's post hoc test for comparison of IL10 to all other control groups. Data (a-d) are representative of 3 independent experiments.

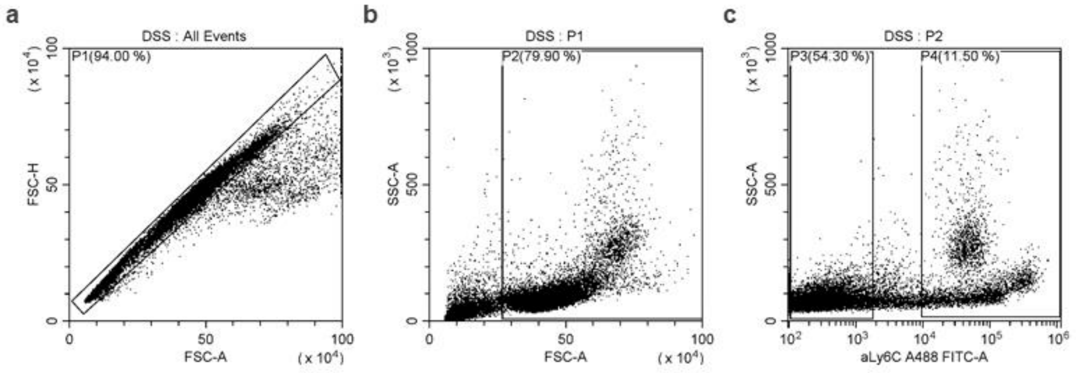

***Supplementary Figure 2*. Gating strategy for sorting splenic Ly6c⁺ and Ly6c⁻ leukocytes**

(a) Single cells gating, P1, followed by a gate, P2, to exclude debris (b). (c) Ly6c⁻, P3, and Ly6c⁺ gating, P4. Data (a-c) corresponds to Figure 2g. Data (a-c) are representative of 3 independent experiments.

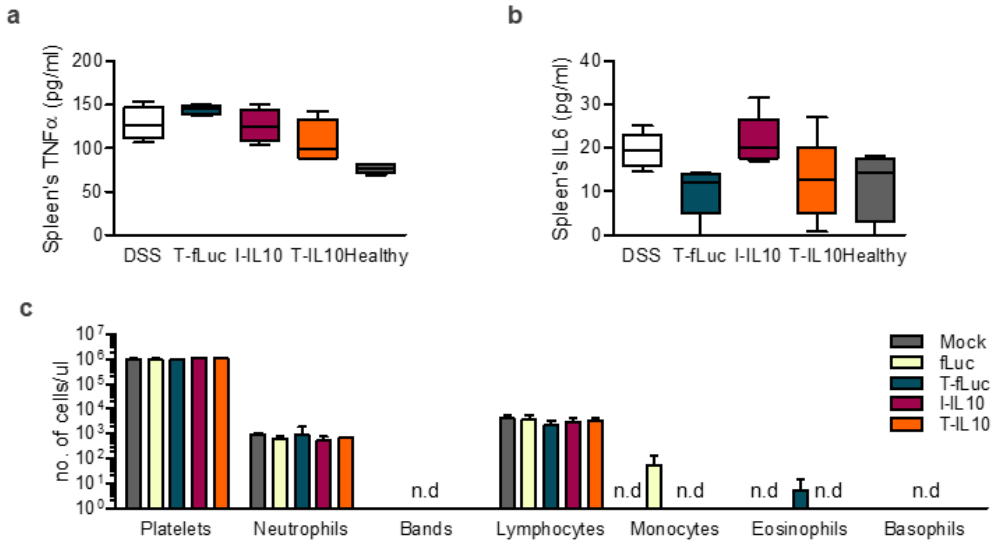

Supplementary Figure 3. **Immunogenicity evaluation by splenic cytokines**

After beginning oral DSS, mice were injected intravenously with αLy6C or isotype control tLNP encapsulating IL10 or fLuc mmRNAs on days 3, 6, and 9 and were sacrificed on day 10. Splenic TNFα (**a**) and IL-6 (**b**) levels in whole tissue lysates were evaluated by ELISA. (**c**) mmRNA LNPs safety as assessed 24 hours after i.v injection by a complete blood count. Data are representative of 3 independent experiments as biological replicates. Data are IQR with a median center line and min to max error bars (a-b) and mean ± s.d. (c), n = 4, * denote $p < 0.05$, ** denote $p < 0.01$, *** denote $p < 0.001$, one-way ANOVA with Dunnett's multiple comparison post hoc test to compare DSS group with all other control groups.

Supplementary Table 1: Characterization of LNP by Dynamic Light Scattering.

	**LNPs**	**TLNPs**
**Hydrodynamic diameter (nm) ± s.d.**	63.71 ± 1.59	65.59 ± 3.37
**ζ potential (mV) ± s.d.**	0.9 ± 0.28	0.5 ± 0.42

Data presented are mean ± s.d. of five independent preparations.

Supplementary Table 1: Statistical analysis for figure 1

		**Mean ± s.e.m.**	**N**	**DF**	**T**	**R^2**
**Figure 1c**	fLuc	101.06 ± 1.65	N = 5			
	IL10	104.88 ± 3.91				
**Figure 1d**	Mock	6.500 ± 2.533	N = 5	Df = 8	T = 20.16	R^2 = 0.9855
	fLuc	3575 ± 176.9				
**Figure 1e**	fLuc	1692 ± 2.505	N = 5	Df = 8	T = 84.39	R^2 = 0.9992
	IL10	3648 ± 23.05				
**Figure 1g**	T-fLuc	101.46 ± 4.13	N = 5			
	T-IL10	101± 2.02				

Supplementary Table 2: Statistical analysis for figure 2

			Mean ± s.e.m.	N	DF	T	R^2
**Figure 2a**	Liver	I-fLuc	9.038e+006 ± 881619	N = 8	Df = 14	T = 8.289	R^2 = 0.8307
		T-fLuc	1.138e+006 ± 361952				
	Spleen	I-fLuc	92163 ± 10311	N = 8	Df = 14	T = 2.245	R^2 = 0.2647
		T-fLuc	459500 ± 163318				
	Intestine	I-fLuc	9918 ± 2315	N = 8	Df = 14	T = 4.707	R^2 = 0.6128
		T-fLuc	689250 ± 144298				
**Figure 2g**	Ly6C-	I-fLuc	19.09 ± 1.441	N = 10	Df = 18	T = 1.548	R^2 = 0.1175
		T-fLuc	24.88 ± 3.451				
	Ly6C+	I-fLuc	9.180 ± 0.8388	N = 10	Df = 18	T = 9.159	R^2 = 0.8233
		T-fLuc	2051 ± 222.9				
**Figure 2h**	Liver	T-fLuc	768.0 ± 38.55	N = 5	Df = 8	T = 4.362	R^2 = 0.7040
		T-IL10	1109 ± 67.98				

	Spleen	T-fLuc	449.3 ± 28.72	N = 5	Df = 8	T = 6.676	R^2 = 0.8813
		T-IL10	688.7 ± 21.47				
	Colon	T-fLuc	136.5 ± 25.2	N = 5	Df = 8	T = 3.353	R^2 = 0.5843
		T-IL10	312.6 ± 46.06				

Supplementary Table 3: Statistical analysis for figure 3

		**PV**	**F**	**R^2**
**Figure 3a**	AST	PV = 0.1703<br>Ns	F = 1.858	R^2 = 0.3313
	ALT	PV=0.2450<br>Ns	F = 1.526	R^2 = 0.2892
	ALP	PV = 0.2375<br>Ns	F = 1.567	R^2 = 0.3092
**Figure 3c**	Platelets	PV = 0.346<br>ns	F = 1.219	R^2 = 0.2583
	Neutrophils	PV = 0.7758<br>ns	F = 0.4429	R^2 = 0.1123
	Lymphocytes	PV = 0.3302<br>ns	F = 1.264	R^2 = 0.2653
**Figure 3d**	Monocytes	PV = 0.1466<br>ns	F = 2.021	R^2 = 0.3660
	Eosinophils	PV = 0.4791<br>ns	F = 0.9211	R^2 = 0.2083

Supplementary Table 4: Statistical analysis for figure 4

	**F**	**R^2**	**Dunnett's comparison**	**Mean difference**	**q**	**Summary**
**Figure 4c**	F = 22.86	R^2 = 0.7010	T-IL10 vs. I-IL10	M.D = 142.0	q = 4.190	***
**Figure 4d**	F = 56.61	R^2 = 0.9188	T-IL10 vs. I-IL10	M.D = -298.6	q = 6.103	***
**Figure 4e**	F=31.26	R^2 = 0.7576	I-IL10 vs. T-IL10	M.D = 295.8	q = 6.960	***
**Figure 4f**	F = 19.21	R^2 = 0.6577	T-IL10 vs. I-IL10	M.D = 6.246	q = 3.029	*
**Figure 4g**	F = 80.22	R^2 = 0.8892	T-IL10 vs. I-IL	M.D = 1.106	q = 5.990	***
