# Supplementary material for: Cell specific delivery of modified mRNA expressing therapeutic proteins to leukocytes
Source: Nat Commun. 2018 Oct 29;9:4493. doi: 10.1038/s41467-018-06936-1 (PMC6206083; doi:10.1038/s41467-018-06936-1)
Supplement: Supplementary file 3 — Reporting Summary [file 41467_2018_6936_MOESM3_ESM.pdf]

naturerearch

Corresponding author(s): Dan Peer

Reporting Summary

Nature Research wishes to improve the reproducibility of the work that we publish. This form provides structure for consistency and transparency in reporting. For further information on Nature Research policies, see Authors & Referees and the Editorial Policy Checklist.

Statistical parameters

When statistical analyses are reported, confirm that the following items are present in the relevant location (e.g. figure legend, table legend, main text, or Methods section).

n/a	Confirmed	
☐	☒	The exact sample size (*n*) for each experimental group/condition, given as a discrete number and unit of measurement
☐	☒	An indication of whether measurements were taken from distinct samples or whether the same sample was measured repeatedly
☐	☒	The statistical test(s) used AND whether they are one- or two-sided<br>*Only common tests should be described solely by name; describe more complex techniques in the Methods section.*
☐	☒	A description of all covariates tested
☐	☒	A description of any assumptions or corrections, such as tests of normality and adjustment for multiple comparisons
☐	☒	A full description of the statistics including central tendency (e.g. means) or other basic estimates (e.g. regression coefficient) AND variation (e.g. standard deviation) or associated estimates of uncertainty (e.g. confidence intervals)
☐	☒	For null hypothesis testing, the test statistic (e.g. *F*, *t*, *r*) with confidence intervals, effect sizes, degrees of freedom and *P* value noted<br>*Give P values as exact values whenever suitable.*
☒	☐	For Bayesian analysis, information on the choice of priors and Markov chain Monte Carlo settings
☒	☐	For hierarchical and complex designs, identification of the appropriate level for tests and full reporting of outcomes
☒	☐	Estimates of effect sizes (e.g. Cohen's *d*, Pearson's *r*), indicating how they were calculated
☐	☒	Clearly defined error bars<br>*State explicitly what error bars represent (e.g. SD, SE, CI)*

Our web collection on statistics for biologists may be useful.

Software and code

Policy information about availability of computer code

Data collection	*Provide a description of all commercial, open source and custom code used to collect the data in this study, specifying the version used OR state that no software was used.*
Data analysis	*Provide a description of all commercial, open source and custom code used to analyse the data in this study, specifying the version used OR state that no software was used.*

For manuscripts utilizing custom algorithms or software that are central to the research but not yet described in published literature, software must be made available to editors/reviewers upon request. We strongly encourage code deposition in a community repository (e.g. GitHub). See the Nature Research guidelines for submitting code & software for further information.

Data

Policy information about availability of data

All manuscripts must include a data availability statement. This statement should provide the following information, where applicable:
- Accession codes, unique identifiers, or web links for publicly available datasets
- A list of figures that have associated raw data
- A description of any restrictions on data availability

All relevant data are available from the authors upon reasonable request.

April 2018

Field-specific reporting

Please select the best fit for your research. If you are not sure, read the appropriate sections before making your selection.

- [x] Life sciences
- [ ] Behavioural & social sciences
- [ ] Ecological, evolutionary & environmental sciences

For a reference copy of the document with all sections, see nature.com/authors/policies/ReportingSummary-flat.pdf

Life sciences study design

All studies must disclose on these points even when the disclosure is negative.

Sample size	The sample size in each experiment was determined to give an appropriate power of the test
Data exclusions	No data was excluded
Replication	All attempts at replication were successful as determined using a statistical analysis
Randomization	Allocation was random in all of the experiments
Blinding	All experiments were conducted in a double blinded fashion in which the researchers were blinded to group allocation and treatments.

Reporting for specific materials, systems and methods

Materials & experimental systems

n/a	Involved in the study	
☒	☐	Unique biological materials
☐	☒	Antibodies
☐	☒	Eukaryotic cell lines
☒	☐	Palaeontology
☐	☒	Animals and other organisms
☒	☐	Human research participants

Methods

n/a	Involved in the study	
☒	☐	ChIP-seq
☒	☐	Flow cytometry
☒	☐	MRI-based neuroimaging

Antibodies

Antibodies used	αLy6c (clone Monts1, BioXcell, USA), Isotype control (clone 2A3, BioXcell, USA), αLy6C (clone HK1.4, BioLegend, USA)
Validation	αLy6c (clone Monts1, BioXcell, USA) and Isotype control (clone 2A3, BioXcell, USA) were purified using protein G and the purity was validated using LAL assay and SDS-Page.<br>αLy6C (clone HK1.4, BioLegend, USA) was validated by the manufacturer and quality control tested by immunofluorescent staining with flow-cytometric analysis

Eukaryotic cell lines

Policy information about cell lines

Cell line source(s)	ATCC
Authentication	All cell lines were purchased directly from ATCC. The authentication of the cell lines was verified using prevalent standards of gene expression using PCR and Flow-Cytometry.
Mycoplasma contamination	All cell lines were routinely checked every two months for Mycoplasma contamination using EZ-PCR™ Mycoplasma Test Kit (Biological Industries, Israel) by manufacture protocol
Commonly misidentified lines<br>(See ICLAC register)	*Name any commonly misidentified cell lines used in the study and provide a rationale for their use.*

Animals and other organisms

Policy information about studies involving animals; ARRIVE guidelines recommended for reporting animal research

Laboratory animals	12 weeks old female C57BL/6 mice (Harlan laboratories, Israel)
Wild animals	The study didn't involve wild animals
Field-collected samples	The study didn't involve field collected samples
